# Heart Failure in Polycystic Ovarian Syndrome and Hypothyroidism: A Retrospective Large Database Analysis

**DOI:** 10.7759/cureus.85281

**Published:** 2025-06-03

**Authors:** Evidence E Ohikhuai, Ngozi T Akueme, Seye Olaniyi, Oluwatosin B Iyun, Remil Thomas, Chidinma J Kanu, Bernard Wiredu, Tanvi Narendrula, Okelue E Okobi

**Affiliations:** 1 Public Health, Jackson State University School of Public Health, Jackson, USA; 2 Dermatology, University of Medical Sciences (UNIMED), Ondo, NGA; 3 Medicine, Princess Alexandra Hospital, Harlow, GBR; 4 Public Health and Family Medicine, University of Cape Town, Cape Town, ZAF; 5 Internal Medicine, Nuvance Health, Vassar Brothers Medical Center, Poughkeepsie, USA; 6 Obstetrics and Gynecology, Richmond Gabriel University, Belair, VCT; 7 Internal Medicine/Oncology, Saint James School of Medicine, Park Ridge, USA; 8 Biology, Marquette High School, Chesterfield, USA; 9 Family Medicine, IMG Research Academy and Consulting LLC, Homestead, USA; 10 Family Medicine, Larkin Community Hospital Palm Springs Campus, Hialeah, USA; 11 Family Medicine, Lakeside Medical Center, Belle Glade, USA

**Keywords:** cardiovascular outcomes, hypothyroidism, inhospital outcomes, mortality, polycystic ovarian syndrome (pcos)

## Abstract

Introduction

Polycystic ovarian syndrome (PCOS) carries similar risks to metabolic syndromes, and the population with hypothyroidism and concurrent PCOS may demonstrate exponential cardiovascular risk. There is a paucity of data on the cardiovascular outcomes of the population with PCOS. We aimed to address this gap.

Methods

We queried the National Inpatient Sample Database (2016-2020), selected the population with a diagnosis of PCOS, and stratified them according to the presence or absence of hypothyroidism. Multivariable logistic regression models were applied to predict the outcomes. The primary outcome was mortality, while secondary outcomes were heart failure (HF), type 2 diabetes mellitus (T2DM), chronic kidney disease (CKD), stroke, hypertension (HTN), hyperlipidemia (HLD), and deep venous thrombosis (DVT).

Results

There were 78,470 hospitalizations with PCOS, and 14.6% (11,455) had hypothyroidism (36.3 years ± 10). Compared to the non-hypothyroid group, the population with hypothyroidism did not differ in terms of mortality (OR: 0.9; 95% CI: 0.6-1.4), HTN (OR: 1.04; 95% CI: 0.9-1.09), stroke (OR: 1.1; 95% CI: 0.8-1.4), or DVT (OR: 0.9; 95% CI: 0.7-1.3) (p > 0.05 for each). However, they had a higher likelihood of HF (OR: 1.2; 95% CI: 1.04-1.4), CKD (OR: 1.2; 95% CI: 1.02-1.3), T2DM (OR: 1.2; 95% CI: 1.1-1.3), and HLD (OR: 1.2; 95% CI: 1.2-1.3) (p < 0.05 for each).

Conclusion

Among the population with PCOS, those with concurrent hypothyroidism had a higher association with HF, CKD, T2DM, and HLD. Risk stratification, prompt screening, close follow-up, and management may improve outcomes in this population.

## Introduction

An increased risk of cardiovascular disease (CVD) has been observed in women who have been diagnosed with polycystic ovarian syndrome (PCOS) [1]. These women also exhibit a higher prevalence of various modifiable risk factors such as diabetes, dyslipidemia, hypertension (HTN), abdominal adiposity, obesity, smoking, heightened alcohol consumption, depression, anxiety, and perceived stress. These factors are associated with a greater likelihood of developing cardiometabolic events [1].

The thyroid gland directly impacts human metabolism, particularly cardiovascular metabolism [2]. Hypofunction of the thyroid gland, ultimately leading to hypothyroidism, affects 3% of persons globally [3]. PCOS impacts 8-13% of women in their reproductive years, with about 70% of those affected being undiagnosed worldwide [4].

Hypothyroidism is known to exert direct negative chronotropic and inotropic impacts on the myocardium, resulting in a diminished heart rate and impaired contractile function, thereby compromising systemic circulation [5]. Concurrent PCOS contributes to an enhanced cardiovascular risk profile, predominantly through the pathophysiology of insulin resistance, which predisposes individuals to adiposity, glucose intolerance, and type 2 diabetes mellitus (T2DM), all factors that amplify cardiac workload and stress. The convergence of these abnormalities can catalyze the development of heart failure (HF) and other poor cardiovascular outcomes [6,7]. 

Emerging evidence suggests that oxidative stress, sustained systemic inflammation, and direct myocardial impairment are additional mechanistic pathways by which these conditions may promote cardiac dysfunction [8]. However, the cardiovascular profile of the PCOS population with and without hypothyroidism is lacking. Given the surmounting data correlating these endocrine disorders with cardiac morbidity, this research endeavor is concentrated on delineating the epidemiological linkage and mechanistic interplay of hypothyroidism within the hospitalized PCOS patient cohort and HF and other cardiovascular outcomes. With the era of artificial intelligence and machine learning, using large datasets like this study presents the opportunity for risk stratification and early detection, which may provide better outcomes [9].

## Materials and methods

This study has been reported following the Strengthening the Reporting of Observational Studies in Epidemiology (STROBE) guidelines [10].

Database

The data used in the study was obtained from the US National Inpatient Sample Database, which covers the period from 2016 to 2020. The National Inpatient Sample Database comprises information about hospital stays across the United States based on billing data submitted by hospitals to statewide data organizations. This database represents more than 97% of the US population [11]. A weighted dataset is generated annually, comprising a 20% stratified sample of patient discharges from community hospitals in the United States. This sampling excludes long-term acute care hospitals and rehabilitation centers. The dataset is designed to achieve national estimates. The 2016-2020 datasets are coded using the International Classification of Diseases, Tenth Revision, and Clinical Modification/Procedure Coding System (ICD-10-CM/PCS). In the NIS, diagnoses are categorized as principal or secondary. This study's principal diagnosis refers to the main ICD-10 code assigned to the hospitalization, while secondary diagnoses comprise any other ICD-10 codes (see Appendices). ICD-10 codes used in our study have been previously validated in other studies [12,13].

Study population and criteria

We performed a retrospective cohort study of hospitalizations from 2016 to 2020 with a principal or secondary diagnosis of PCOS and a secondary diagnosis of hypothyroidism. We used ICD-10 codes obtained from other studies with validated ICD-10 codes [14,15]. This study was exempted from the Institutional Review Board approval because it utilized de-identified data available online at www.hcup-us.ahrq.gov. 

From 2016 to 2020, hospitalizations related to PCOS were analyzed for the study population. The study parameters comprised sociodemographic characteristics, medical comorbidities, hospital attributes, and primary and secondary outcomes. We excluded patients aged <18 years. Baseline hospitalization characteristics for PCOS with and without a secondary diagnosis of hypothyroidism were studied.

Data analysis

We selected the population with a diagnosis of PCOS and stratified them according to the presence or absence of hypothyroidism. Then, the chi-squared test and Student's t-test were used to compare categorical and continuous variables. Continuous variables were represented as mean + standard deviation (SD), while categorical variables were represented as percentages. When comparing variables or groups within this data, we assumed that they were independent of each other and had enough statistical power. If this assumption was false, we used Fisher's exact test instead of the chi-squared test. Similarly, if the data was not normally distributed, we replaced Student's t-test with the Mann-Whitney U test. For trends in hospitalization, we utilized the Cochran-Armitage trend analysis to assess the proportion of this population of interest, i.e., hypothyroidism, over the study period, i.e., "YEARS".

Multivariable logistic regression models were applied to account for patient- and hospital-level confounders and to predict the outcomes. We modeled the dependent variable as the outcomes of interest while using independent variables as predictors. We also included their baseline characteristics to account for patient- and hospital-level confounders. This model allowed for the estimation of outcomes using a two-sided p-value and confidence interval (CI). A p-value of ≤0.05 was the statistical threshold of significance. All statistical analyses were conducted using the SAS 9.4 software (SAS Inc., Cary, NC, USA).

Outcomes of interest

Our primary outcome was mortality, while secondary outcomes were T2DM, HF, chronic kidney disease (CKD), stroke, HTN, hyperlipidemia (HLD), and deep venous thrombosis (DVT). 

We also compared the median cost of hospitalization, mean length of hospitalization (length of stay (LOS)), and trend of hospitalization between both groups.

## Results

Baseline characteristics

During the study period, a total of 78,470 hospitalizations were identified with a diagnosis of PCOS, of which 14.6% (11,455 patients) had concurrent hypothyroidism.

Regarding racial distribution, the majority of PCOS patients were White (67.2%), followed by Hispanic (12.7%), Black (12.1%), Asian/Pacific Islander (4%), and Native American (0.6%). When stratified by hypothyroidism status, 77% of patients in the hypothyroid group were White, compared to 65.6% in the non-hypothyroid group (χ²(5) = 105.32; p < 0.01; Cramér's V = 0.11), highlighting a significant racial discrepancy.

Notably, obesity (BMI >30 kg/m²) was highly prevalent in both groups, affecting 52.3% of hypothyroid patients compared to 47.7% of non-hypothyroid patients, indicating a significant association (χ²(1) = 6.43; p = 0.01; Cramér's V = 0.03). Psychiatric comorbidities were also more frequent in the hypothyroid group, with generalized anxiety disorder affecting 32.6% vs. 27.3% (χ²(1) = 8.72; p < 0.01; Cramér's V = 0.04) and major depressive disorder affecting 25.1% vs. 21.4% (χ²(1) = 7.51; p < 0.01; Cramér's V = 0.03).

Other significant differences included the prevalence of HTN (22.3% vs. 17.5%; χ²(1) = 18.75; p < 0.01; Cramér’s V = 0.05) and smoking rates (19.2% vs. 21.6%; χ²(1) = 9.21; p < 0.01; Cramér's V = 0.04) (Table 1).

**Table 1:** Baseline characteristics of the PCOS population with and without hypothyroidism. All representations are numbers (n) and their corresponding percentages (%) except mean age with standard deviation. PCOS: polycystic ovarian syndrome

Characteristics	No hypothyroidism (N (%))	Hypothyroidism (N (%))	P-value
	67,015 (85.4)	11,455 (14.6)	
Mean age	32.8 (SD = 9)	36.3 (SD = 10)	0.68
Race			
White	42,730 (65.6)	8,560 (77.0)	0.01
Black	8,670 (13.3)	540 (4.9)	
Hispanic	8,705 (13.3)	995 (9.0)	
Asian or Pacific Islander	2,410 (3.7)	605 (5.4)	
Native American	360 (0.6)	75 (0.7)	
Others	2,300 (3.5)	345 (3.1)	
Insurance type			
Medicare	3,625 (5.4)	1,010 (8.8)	0.08
Medicaid	16,580 (24.8)	2,080 (18.2)	
Private insurance	41,965 (62.7)	7,640 (66.8)	
Self-pay	2,510 (3.8)	340 (3.0)	
No charge	140 (0.2)	20 (0.2)	
Others	2,110 (3.2)	345 (3.0)	
Median household income			
0-25th percentile	16,245 (24.4)	2,430 (21.5)	0.15
26th to 50th percentile (median)	18,080 (27.2)	3,010 (26.6)	
51st to 75th percentile	17,160 (25.8)	2,930 (25.9)	
76th to 100th percentile	15,000 (22.6)	2,960 (26.1)	
Region of the hospital			
Northeast	4,125 (15.8)	650 (13.3)	0.20
Midwest	6,435 (29.1)	1,425 (29.1)	
South	11,400 (43.5)	2,010 (41.0)	
West	4,230 (16.2)	815 (16.6)	
Location/teaching status of the hospital			
Rural	4,045 (6.0)	735 (6.4)	0.12
Urban nonteaching	10,225 (15.3)	1,700 (14.8)	
Urban teaching	52,745 (78.7)	9,020 (78.7)	
Comorbidities			0.40
Coronary artery disease	1,095 (1.6)	240 (2.1)	0.10
Atrial fibrillation	640 (1.0)	145 (1.3)	0.02
Myocardial infarction	265 (0.4)	65 (0.6)	0.01
Inflammatory bowel disease	530 (0.8)	75 (0.7)	0.10
Pulmonary hypertension	440 (1.0)	115 (1.0)	<0.01
Hypertension	11,715 (17.5)	2,550 (22.3)	<0.01
Acute kidney injury	2,540 (4.0)	575 (5.0)	0.3
Respiratory failure	2,980 (4.5)	720 (6.3)	0.01
Chronic obstructive pulmonary disease	1,065 (1.6)	295 (2.6)	0.03
Major depressive disorder	14,365 (21.4)	2,875 (25.1)	0.01
Bipolar affective disorder	3,780 (5.6)	750 (6.6)	0.7
Generalized anxiety disorder	18,320 (27.3)	3,730 (32.6)	<0.001
Smoking	14,445 (21.6)	2,195 (19.2)	<0.001
Breast cancer	165 (0.3)	50 (0.4)	0.2
Current chronic long-term steroid use	555 (0.8)	130 (1.1)	0.5
Obesity	31,980 (47.7)	5,985 (52.3)	0.01
Pulmonary embolism	805 (1.2)	95 (0.8)	0.04
Inpatient procedures			
Percutaneous coronary intervention	115 (0.2)	15 (0.1)	0.4
Blood transfusion	1,500 (2.2)	295 (2.6)	0.2

Outcomes

Compared to the non-hypothyroid group, the population with hypothyroidism exhibited a significantly higher likelihood of HF, CKD, T2DM, and HLD, with odds ratios of 1.2 (95% CI: 1.04-1.4) for HF, 1.2 (95% CI: 1.02-1.3) for CKD, 1.2 (95% CI: 1.1-1.3) for T2DM, and 1.2 (95% CI: 1.2-1.3) for HLD (p < 0.05 for each). Chi-squared analyses revealed statistically significant differences in prevalence between the groups, with test statistics of χ²(1) = 7.02 and p = 0.008 for HF, χ²(1) = 6.89 and p = 0.009 for CKD, χ²(1) = 8.44 and p = 0.004 for T2DM, and χ²(1) = 9.51 and p = 0.003 for HLD, indicating a small but meaningful effect size (Cramér's V ranging from 0.03 to 0.04).

Additionally, hypothyroidism was associated with a longer length of hospitalization (four vs. 3.6 days) and increased hospitalization costs ($43,509 vs. $39,943; p < 0.001), with chi-squared results demonstrating statistical significance (χ²(1) = 12.65 and p < 0.001 for LOS and χ²(1) = 15.78 and p < 0.001 for cost) and corresponding effect sizes (Cramér's V = 0.05-0.06). These findings emphasize the significant impact of hypothyroidism on hospitalization burden and cardiovascular/metabolic outcomes among patients with PCOS (Table 2).

**Table 2:** Length of hospitalization. Values are represented as the mean.

	Hypothyroidism	No hypothyroidism	P-value
Length of hospitalization	4 days	3.6 days	<0.001
Cost of hospitalization	$43,509	$39,943	<0.001

**Figure 1:**
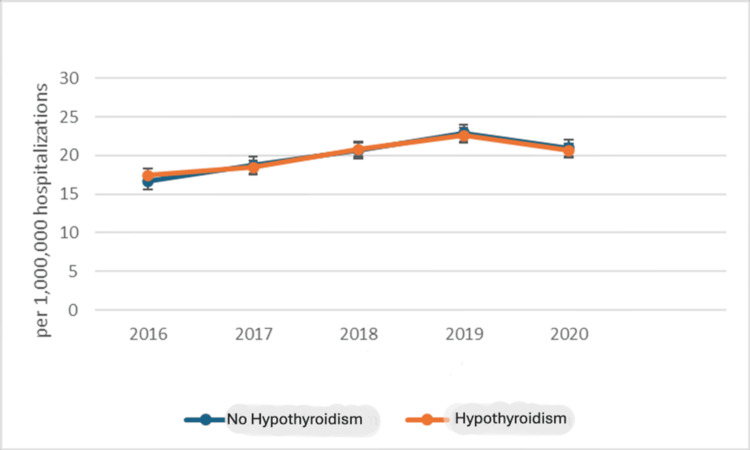
Trend analysis of hospitalization of the polycystic ovarian syndrome population stratified according to the presence or absence of hypothyroidism.

A Cochran-Armitage trend test was performed to assess changes in the proportion of hypothyroidism among PCOS hospitalizations over the study period. The results indicated that the variation in hypothyroidism prevalence across the years was not statistically significant (χ²(1) = 1.02; p = 0.31), confirming a stable trend (Figure 1).

The chi-squared analysis comparing racial distribution between PCOS patients with and without hypothyroidism demonstrated a statistically significant difference (χ²(5) = 105.32; p < 0.01; Cramér's V = 0.11), highlighting a notable racial discrepancy. White patients had a higher prevalence of hypothyroidism (76.98%) compared to Black (4.86%) and Hispanic (8.95%) patients, which may suggest demographic variations in endocrine risk factors (Table 3).

**Table 3:** The prevalence of hypothyroidism and PCOS according to race and ethnicity. Values are represented as percentages (%) and numbers (n). PCOS: polycystic ovarian syndrome

	White % (n)	Black % (n)	Hispanic % (n)	Asian/Pacific Islander % (n)	Native American % (n)	Others % (n)	P-value
No hypothyroidism	65.56 (42,730)	13.30 (8,670)	13.36 (8,705)	3.70 (2,410)	0.55 (360)	3.53 (2,300)	<0.01
Hypothyroidism	76.98 (8,560)	4.86 (540)	8.95 (995)	5.44 (605)	0.67 (75)	3.10 (345)	<0.01
PCOS prevalence by race	67.23 (51,290)	12.07 (9,210)	12.71 (9,700)	3.95 (3,015)	0.57 (435)	3.47 (2,645)	<0.01

## Discussion

**Figure 2:**
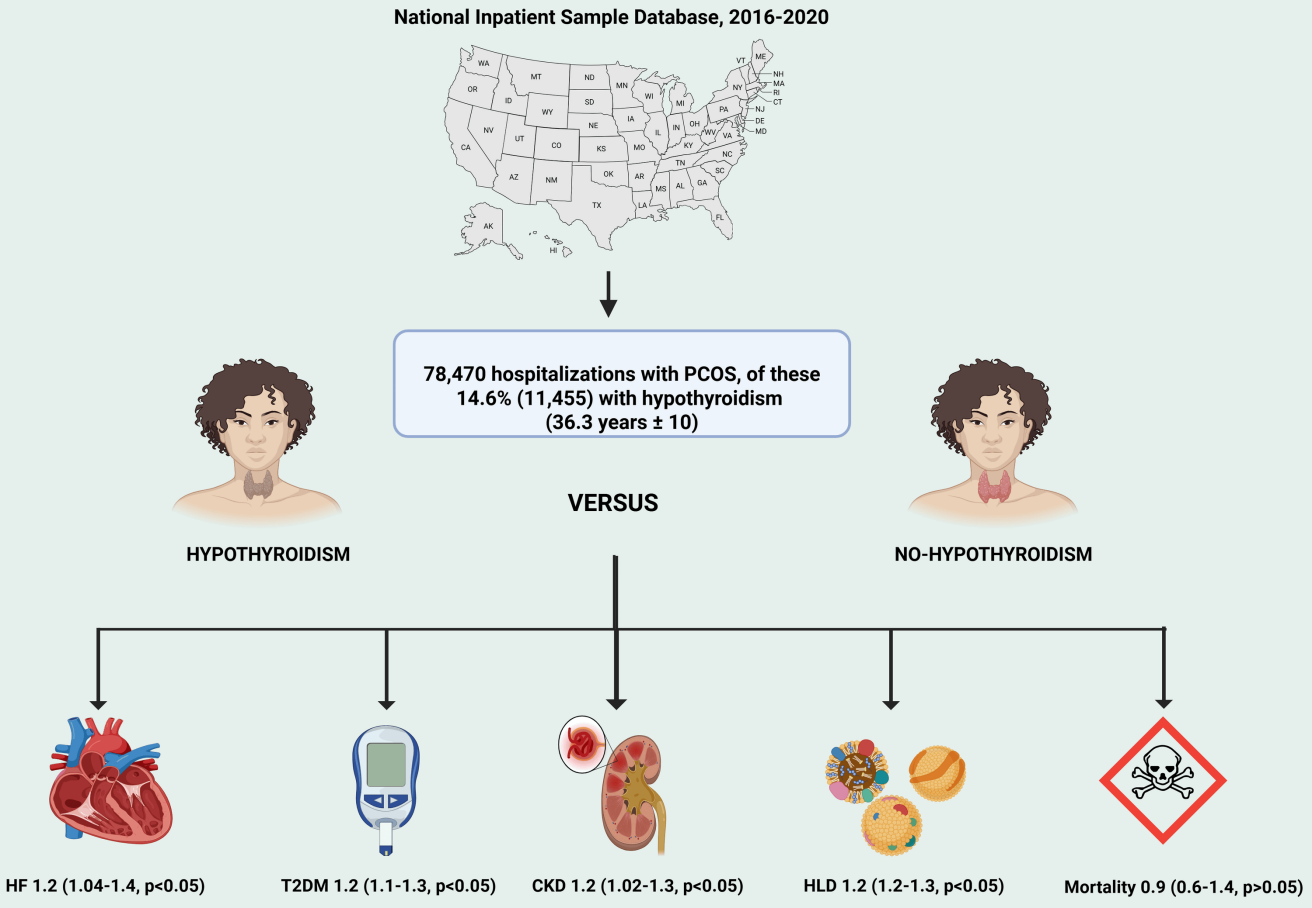
Graphical abstract comparing the population with PCOS with and without hypothyroidism. PCOS: polycystic ovarian syndrome; HF: heart failure; CKD: chronic kidney disease; T2DM: type 2 diabetes mellitus; HLD: hyperlipidemia This figure is an original creation of the authors.

This study had intriguing results. It demonstrated that among 78,470 hospitalizations with PCOS, those with concurrent hypothyroidism had a higher likelihood of HF, CKD, T2DM, and HLD. However, there was no difference in terms of mortality, HTN, stroke, and DVT in the hypothyroid vs. non-hypothyroid group (Figure 2).

These findings suggest an association between PCOS, hypothyroidism, T2DM, and HLD, consistent with recent international studies [16,17]. In one study, 52 euthyroid and 48 subclinical hypothyroidism (SCH) research participants were identified from a hospital-based cross-sectional study conducted among 100 PCOS patients. It was observed that the Homeostatic Model Assessment for Insulin Resistance (HOMA-IR) and fasting serum insulin were significantly elevated in the SCH group compared to the euthyroid group [17]. This may result from the complex relationship between insulin resistance indices and thyroid-stimulating hormone (TSH) levels, explained by the tendency of hypothyroidism to increase adiposity and pro-inflammatory markers [17]. PCOS, obesity, thyroid dysfunction, and autoimmunity interact uniquely to create a complicated pathophysiological pathway that intensifies insulin resistance and raises the metabolic and cardiovascular risk in PCOS-affected women [18]. A meta-analysis of 28 studies also demonstrated higher insulin resistance in individuals with SCH, which is intriguing as insulin resistance is a central pathogenic feature of PCOS [19]. Another study revealed its unfavorable effects on high-density lipoprotein (HDL)-cholesterol and triglycerides (TG) [20].

Several studies have also shown an increased likelihood of HF and CKD in PCOS patients with coexisting hypothyroidism [18,21]. Even in the context of normal TSH levels, the hypothyroid state, precisely a low triiodothyronine level, has been linked to a poor prognosis and diminished cardiac performance in HF. Low levels of thyroid hormones alter cardiac gene expression and increase systemic vascular resistance, both resulting in a reduction of cardiac contractility and cardiac output [22]. When compared to 30 euthyroid control patients, a group of 54 individuals with SCH and elevated TSH had adverse measurements of multiple diastolic and systolic left ventricular function, assessed by echocardiography [23]. The increased likelihood for the development of CKD in PCOS patients with concurrent hypothyroidism could be explained by PCOS and hypothyroidism being independently associated with T2DM, which, when poorly controlled, can lead to diabetic nephropathy, which is known to be the most common cause of end-stage renal disease (ESRD) in the United States [24]. A prospective follow-up of 480 euthyroid patients and 89 patients with SCH for 26 months revealed adverse renal outcomes in the SCH group, with increased hazard ratios (HRs) for a composite outcome incorporating doubling of serum creatinine (SCr) and ESRD [25].

Previous studies that have examined the effect of PCOS on vascular diseases like HTN, DVT, and stroke showed increased odds of these diseases in the hypothyroid group compared to the non-hypothyroid group [26]. This could be explained by the effect of PCOS with concurrent hypothyroidism on lipid profile. Women with PCOS are prone to dyslipidemia. The most common dyslipidemia features in PCOS patients are hypertriglyceridemia, decreased HDL-cholesterol concentrations, and small, dense low-density lipoprotein (LDL) particles characteristic of the atherogenic lipoprotein phenotype [27]. Therefore, it is speculated that PCOS patients have a higher tendency to develop atherosclerosis. Atherosclerosis drives the narrowing of the blood vessels, increasing total peripheral resistance and blood pressure. Reduction of the luminal diameter of the blood vessels also reduces blood flow to different organs like the brain and limbs, resulting in ischemia, as seen in stroke. Deposition of LDL particles in the lumen of blood vessels causes oxidative stress and subsequent endothelial injury [28]. LDL are thrombogenic particles that cause the recruitment of platelets, and all these contribute to the narrowing of the blood vessels and, hence, stasis, which increases the likelihood of DVT [27,29].

Contrary to initial expectations, this study did not reveal significant differences regarding HTN, stroke, DVT, and overall mortality. This was in keeping with a few other observational studies, one of which enrolled 3,078 subjects with and without hypothyroidism and demonstrated no significant differences in office systolic and diastolic blood pressure measurements [30]. Furthermore, although we sought to compare both arms (hypothyroidism and no hypothyroidism), the population with PCOS is at baseline already predisposed to obesity, T2DM, heart disease, and HTN [4]. This may explain the non-difference in some outcomes of this study. Another possibility may be attributed to medical advances in CVD and especially for acute heart diseases, including specialized coronary care units, new pharmaceuticals with improvements in HTN management, the use of statins to lower circulating cholesterol levels, the development and timely use of thrombolysis, and advanced surgical procedures such as bypass surgery and angioplasty [31]. A more active approach to prevention and disease management, and behavioral changes such as reduced smoking rates and improved diet, further diminished the likelihood of heart and vascular diseases and the associated mortality risk [31-33]. The age group being studied may also be of value in this finding. With a mean of 36.3 years, it is unlikely to find an overwhelming difference in mortality, given that age is one of the single most significant variables that impact mortality [34]. We may argue that the accumulation of events resulting from the hypothyroid state may predispose to mortality down the road, but this study cannot draw such conclusions.

Nonetheless, this study has many strengths. Firstly, this population of 78,470 provides one of the largest databases of individuals with PCOS. Secondly, this sample had a decent inclusion of minority races, including Blacks, Hispanics, Asians, and Native Americans, for better generalizability to the real-world scenario. Thirdly, given the relatively young age of this population, which is relatively healthy, these findings are clinically thought-provoking since this population is often not burdened with comorbidities that may confound associations.

Limitations

It should be noted that this study has some limitations due to its retrospective and observational design. We could not obtain granular data such as laboratory values, echocardiogram parameters, levels of serum TSH, antibody titers, or thyroid ultrasound data. Additionally, we could not assess the severity of comorbidities, contraindications to procedures, and patients' beliefs and preferences. Age at diagnosis of hypothyroidism and the time difference between diagnosis of hypothyroidism and HF could not be ascertained; thus, causality cannot be inferred. Furthermore, despite multivariable analysis, confounding factors, such as obesity severity, medication use such as metformin and levothyroxine, or socioeconomic status, may influence outcomes, as could selection bias from the increased evaluation of the PCOS group. Nonetheless, this study holds significant power, involves a relatively large minority population, and adds to the body of knowledge through hypothesis-generating associations that may inform future studies.

## Conclusions

This analysis of a large nationally representative database indicates that the population with PCOS and concurrent hypothyroidism may be at a significantly higher risk of developing HF, CKD, T2DM, and HLD compared to those without hypothyroidism. These findings emphasize the need for a comprehensive approach to patient care, as endocrine disorders and cardiovascular health are closely interrelated. Future studies that may inform guideline recommendations, clinical risk stratification, early detection, and management are warranted. 

## Appendices

**Table 4:** ICD-10 and procedural codes. * signifies all diagnostic codes related to the aforementioned ICD-10 code included in the analysis. ICD-10: International Classification of Diseases, Tenth Revision

Diagnosis/procedure	ICD-10 codes
Hypertension	I10, I11, I119
Hyperlipidemia	E785, E7849, E782, E784
Diabetes mellitus	E11, E119
Myocardial infarction	
ST-segment elevation myocardial infarction	I21, I2111, I219, I210, I2119, I219, I2101, I212, I21A9, I2102, I2121, I22, I2109, I2129, I220, I211, I213, I221, I228, I229
Non-ST-segment elevation myocardial infarction	I214, I222
Acute kidney injury	N17
Chronic kidney disease	N18, N181, N182, N183, N1830, N1831, N1832, N184, N185, N186, N189
Chronic liver disease	K70, K71, K72, K73, K74, K75, K76, K77
Chronic obstructive pulmonary disease	J41, J42, J43, J44
Obesity (BMI >30)	E663, E668, E669, E6601, E6609, E660, E66
Cigarette smoking	F17, F203, F17213, F172, F208, F17218, F1720, F209, F17219, F1200, F1721, F1722, F1201, F17210, F17220, F202, F17211, F17221, F17223, F17228, F17229, F1729, F17290, F17291, F17293, F17298, F17299, Z87891
Alcohol use	F10
Illicit drug use	F11*, F12*, F13*, F14*, F15*, F16*
Steroid use	Z7952, Z92241, G9340
Peripheral vascular disease	I739, I738
Carotid artery disease	I652
Old or previous stroke	I63, I60, I61, I62, I63, T8281
Hypothyroidism	E03*
Anemia	D50, D51, D52, D53, D55, D56, D57, D58, D59, D60, D61, D62, D63, D64
Generalized anxiety disorder	F41*
Major depressive disorder	F32*, F33*
Bipolar affective disorder	F31*
Atrial fibrillation	I48, I480, I481, I4811, I4819, I482, I4820, I4821, I489, I4891
History of respiratory failure	J96, J960, J9600, J9600, J9601, J9602, J961, J9610, J9611, J9612, J962, J9620, J9621, J9622, J969, J9690, J9691, J9692
Pulmonary hypertension	I270, I272, I2720, I2721, I2722, I2723, I2724, I2729
Cardiac arrest	I46, I462, I468, I469
Cardiogenic shock	R570, T8111XA, T8111XS
Pulmonary embolism	I2602, I2609, I2692, I2699
History of right heart failure	I50811, I50813
Heart failure	I50*
Percutaneous coronary intervention	027034*, 02703D*, 02704D*, 02703Z*
Blood transfusion	30230H*, 30230N*, 30230P*, 30233H*
